# Strong coupling superconductivity in a quasiperiodic host-guest structure

**DOI:** 10.1126/sciadv.aao4793

**Published:** 2018-04-13

**Authors:** Philip Brown, Konstantin Semeniuk, Diandian Wang, Bartomeu Monserrat, Chris J. Pickard, F. Malte Grosche

**Affiliations:** 1 Cavendish Laboratory, University of Cambridge, Cambridge, UK.; 2 Department of Physics and Astronomy, Rutgers University, Piscataway, NJ 08854, USA.; 3 Department of Materials Science and Metallurgy, University of Cambridge, Cambridge, UK.; 4 Advanced Institute for Materials Research, Tohoku University, Sendai, Japan.

## Abstract

We examine the low-temperature states supported by the quasiperiodic host-guest structure of elemental bismuth at high pressure, Bi-III. Our electronic transport and magnetization experiments establish Bi-III as a rare example of type II superconductivity in an element, with a record upper critical field of ~ 2.5 T, unusually strong electron-phonon coupling, and an anomalously large, linear temperature dependence of the electrical resistivity in the normal state. These properties may be attributed to the peculiar phonon spectrum of incommensurate host-guest structures, which exhibit additional quasi-acoustic sliding modes, suggesting a pathway toward strong coupling superconductivity with the potential for enhanced transition temperatures and high critical fields.

## Teaser

The quasiperiodic structure of high-pressure bismuth gives rise to unusually strong electron-phonon coupling.

## INTRODUCTION

The periodic nature of crystalline lattices forms the bedrock on which much of modern condensed matter physics is built. This includes the theories of lattice vibrations and of electronic energy bands—which lead on to widespread applications in semiconductor physics and optoelectronics—as well as the theories of superconductivity and magnetism. Conventional lattice periodicity is broken in quasiperiodic materials such as the well-known quasicrystals. The diverse structures of these fully ordered but not periodic materials have been studied widely, but, comparatively, little is known about their vibrational and electronic excitations. It has long been realized [see, for example, the work by Janssen *et al*. (*1*) and references therein] that electronic states in the presence of quasiperiodic potentials can become localized or even critical, in the sense that the wave function displays a power-law decay, and that they can exhibit highly fragmented spectra akin to the Hofstadter butterfly (*2*). The vibrational excitations of quasiperiodic systems, likewise, may exhibit surprising properties, one of which is the emergence of a sliding, or phason mode (*3*, *4*), observed experimentally in the incommensurate chain compound Hg_3-δ_AsF_6_ (*5*). This phenomenon is illustrated in composite structures, in which two interpenetrating sublattices share the same unit cell in two spatial directions but have incommensurate lattice constants in the third direction. Because the total energy of such a system is independent of the relative position of the two sublattices, they can, if pinning is ignored, slide freely with respect to each other, generating an apparent fourth acoustic mode.

**Fig. 1:**
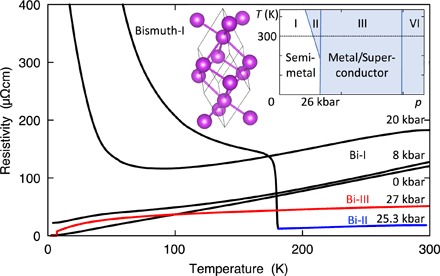
Evolution of the temperature dependence of the resistivity ρ(*T*) of bismuth with pressure *p*. As *p* approaches 25 kbar, ρ rises rapidly at low *T*, indicating a reduction in the carrier concentration. Over a narrow range in *p* and *T* above 25 kbar, Bi is known to assume the Bi-II structure (blue line), which goes along with a drastic decrease in ρ(300 K). At higher pressures still, Bi orders in the incommensurate Bi-III structure (red line). (Inset) Crystal structure of Bi-I and schematic *p*-*T* phase diagram of Bi.

**Fig. 2:**
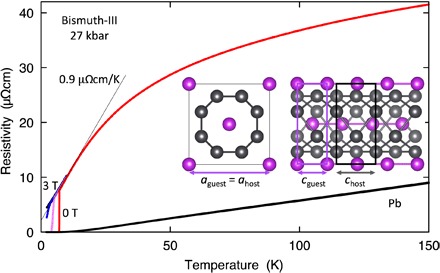
ρ(*T*) for bismuth at 27 kbar (Bi-III), showing a nearly linear *T* dependence at low *T* above a superconducting transition at *T*_c_ ≃ 7.05 K. Moderate magnetic fields (1, 2, and 3 T) suppress *T*_c_, but the critical field ≃ 2.5 T is much higher than that of Pb, which has a similar *T*_c_ ≃ 7.2 K but a far weaker *T* dependence of ρ(*T*) (black line). Left inset: Crystal structure of Bi-III, showing the commensurate arrangement within the *ab* plane of guest (purple) and host atoms (gray). Along the *c* axis (right inset), the discrepancy between the lattice constants of guest and host atoms becomes apparent.

Elemental bismuth offers a fresh perspective on this long-standing problem. Bismuth stands out among the elements for the exceptionally small carrier density of ~ 10^−5^ per atom within its semimetallic ambient pressure structure, the Bi-I phase. Recent reports of topologically protected surface states in Bi_0.91_Sb_0.09_ (*6*), correlated electron effects in high magnetic fields (*7*–*9*), and superconductivity at ultralow temperatures (*10*) demonstrate resurgent interest in this intriguing material. Under applied pressure, bismuth undergoes a cascade of structural transitions into metallic phases with high carrier density: first, into the Bi-II phase, which is metastable around 25 kbar at low *T*, then the Bi-III phase, which extends from about 26 to 80 kbar, and beyond that the Bi-VI phase with some uncertainty about additional intervening Bi-IV and Bi-V phases (inset of Fig. 1) (*11*–*14*). Whereas the Bi-I, Bi-II, and Bi-VI structures are rhombohedral, monoclinic, and body-centered cubic, respectively, the Bi-III phase assumes an incommensurate composite structure, in which a host lattice contains chains of guest atoms aligned along the *c* axis with a lattice constant that is incommensurate with the host unit cell (inset of Fig. 2) (*15*). Bi-III therefore lacks periodicity despite having long-range order. Superconductivity has been reported in all of the abovementioned high-pressure phases of bismuth (*12*), but detailed measurements of the critical field and of normal state transport properties, and their interpretation in the context of the unusual lattice structures involved, have been lacking.

Here, we examine the superconducting and normal state properties of Bi-III and correlate them with results of numerical studies of the electronic structure and vibrational spectrum. We find that (i) Bi-III is one of very few elemental type II superconductors, with a superconducting transition temperature *T*_c_ = 7.05 K and a low-temperature upper critical field of nearly 2.5 T, a record value among the elements; (ii) its normal state resistivity ρ is linear in temperature *T* at low *T* with an unusually high slope; (iii) both the slope *d*ρ/*dT* and the high critical field suggest strong coupling superconductivity with electron-phonon coupling constant λ ~ 2.8; and (iv) these properties can be attributed to the unusual phonon spectrum expected for incommensurate host-guest structures.

## RESULTS

The evolution of the temperature-dependent electrical resistivity ρ(*T*) with pressure *p* is summarized in Fig. 1. With increasing pressure, the resistivity traces change from metallic (*d*ρ/*dT* > 0 at low *T*) to semiconducting, suggesting that the tiny indirect band overlap in semimetallic, ambient pressure Bi is continuously reduced, extrapolating to zero between 20 and 25 kbar (*16*). At room temperature, rhombohedral Bi-I transforms into monoclinic Bi-II over a narrow pressure range at about 25 kbar. As the trace at 25.3 kbar in Fig. 1 shows, the metallic Bi-II structure, which hosts a much higher carrier density than Bi-I and, therefore, exhibits a smaller ρ at 300 K, replaces Bi-I at high *T*; however, on cooling, the sample reverts to Bi-I. On further increasing the pressure above 26 kbar, bismuth transforms to the incommensurate host-guest structure Bi-III, which is accompanied by a step increase in ρ(300 K). Resistivity traces within the Bi-III phase (Fig. 2) show (i) a sharp superconducting transition with a *T*_c_ of ≃ 7.05 K, (ii) a linearly *T*-dependent normal state resistivity at low *T* with an unusually steep gradient ≃ 0.9 μΩcm K^−1^, and (iii) a sublinear, saturating ρ(*T*) above about 20 K. These observations differ markedly from the form of ρ(*T*) in quasicrystals [see, for example, the study by Dolinšek (*17*)], which usually exhibit a nearly constant or even increasing resistivity on cooling. Moreover, the linear form of ρ(*T*) at low *T* and its rapid saturation with increasing *T* contrast strongly with ρ(*T*) of Pb, the immediate neighbor of Bi in the periodic table, which has a similar *T*_c_ ≃ 7.2 K. They are unexpected in a material in which electronic structure calculations show very little admixture of *d* or *f* states near the Fermi energy (*13*), suggesting negligible electronic correlations.

**Fig. 3:**
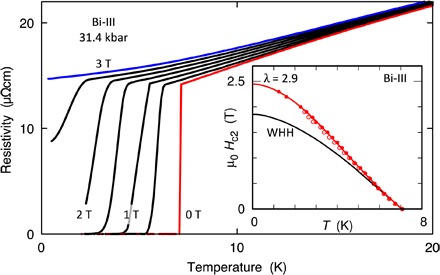
Low *T* resistivity of bismuth at 31.4 kbar in 0.5 T field increments from 0 to 3 T. Inset: Upper critical field *B*_c2_ for Bi-III at 31.4 kbar (full symbols) and 27 kbar (empty symbols), extracted from the mid-point of the resistive transition. The measured data deviate strongly from the weak coupling clean limit Werthamer-Helfand-Hohenberg (WHH) form (*18*) (black line), suggesting a strong coupling description (*19*) (red line for λ = 2.9).

The nature of the pressure-induced superconducting state in Bi-III can be probed further by tracking the superconducting transition in applied magnetic fields (Fig. 3). The temperature dependence of the upper critical field *B*_c2_ (inset of Fig. 3), determined from the midpoint of the resistive transition, deviates from the standard weak-coupling form (*18*). Instead, it agrees much better with a numerical solution of a strong coupling model with a single, low-lying Einstein mode (*19*), if we choose λ = 2.9, close to the value of 2.75 suggested by our analysis of ρ(*T*) following below. In the low-temperature limit, the critical field extrapolates to *B*_c2_(0) ≃ 2.45 T, which corresponds to a superconducting coherence length ξ ≃ 116 Å according to the standard expression *B*_c2_ = Φ_0_/(2πξ^2^), where Φ_0_ = *h*/2*e* is the flux quantum. This gives Bi-III the highest upper critical field of any element, with the possible exception of Li at very high pressures, for which there are conflicting reports (*20*, *21*).

**Fig. 4:**
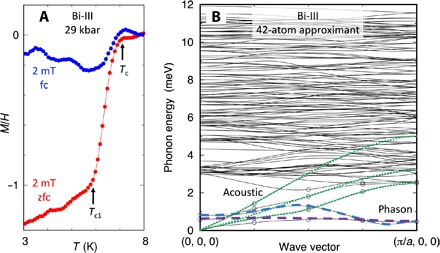
Magnetic and phonon properties of Bi-III. Left: Magnetization *M* over applied field *H* (both in SI units) in Bi at 29 kbar as a function of temperature for μ_0_*H* = 0.002 T, on warming zero-field cooling (zfc) and field cooling (fc). Right: Phonon dispersion computed for wave vectors **q** perpendicular to *c* indicated by open circles, interpolated in between. We identify not only three acoustic modes (dotted lines) and a spaghetti of optical modes but also two further modes at very low energy, which have low dispersion (dashed lines). These correspond to the zero-frequency phason modes expected in the incommensurate structure of Bi-III.

Measurements of the magnetization over a wide pressure range show a superconducting volume fraction of order 1 for zero field cooling (zfc) (Fig. 4A) and a smaller but still sharp drop in the magnetization in field-cooled measurements, consistent with Meissner flux expulsion. This commonly observed reduction with respect to the zfc drop in type II superconductors can be attributed to pinning effects. Whereas the high-temperature onset *T*_c_ of the magnetization step in small fields represents the transition between the mixed and normal states, the foot of the step at *T*_c1_ indicates the transition between the Meissner state and the mixed state. Estimates of the lower critical field *B*_c1_ can be extracted from the field dependence of *T*_c1_, leading to an upper limit for *B*_c1_ of about 12 mT for *p* between 30 and 40 kbar (Supplementary Materials). This corresponds to a lower limit on the penetration depth $\ell_{\lambda }=\xi \sqrt{B_{c2}/(2B_{c1})}$ of about 117 nm and a Ginzburg-Landau parameter κ of at least 10.

Key information about the low *T* electronic and vibrational properties of Bi-III can be derived from the anomalous *T* dependence of its normal state resistivity (Fig. 2). We attribute the linear *T* dependence of ρ(*T*) at low *T* to scattering from low-lying branches of the phonon spectrum, which can be modeled by the Bloch-Grüneisen formula (*22*)

$$\rho (T)=\rho_{0}+\frac{4\pi }{\epsilon_{0}\Omega_{p}^{2}}\sum_{q}\alpha_{q}^{2}T{\left(\partial n_{q}/\partial T\right)}_{\omega_{q}}$$ (1)

The sum is taken over all phonon wave vectors and branches within a suitable cutoff. Ω_p_ = [*ne*^2^/(ϵ_0_*m*)]^1/2^ is the plasma frequency (with *n* the charge carrier concentration and *m* the band mass), $\alpha_{q}^{2}$ is a **q**-dependent Fermi surface average of the electron-phonon interaction weighted for transport calculations, ω_**q**_ represents the phonon dispersion, and *n*_**q**_ = (exp [*ℏ*ω_**q**_/(*k*_B_*T*)] − 1)^−1^ is the Bose occupation number. Phonon modes with *ℏ*ω_**q**_ < *k*_B_*T* contribute a *T*-linear term $\rho_{1}(T)=\frac{4\pi }{\epsilon_{0}\hbar \Omega_{p}^{2}}k_{B}T\sum_{q}\alpha_{q}^{2}\omega_{q}^{-1}$, which can be directly related to the electron-phonon coupling constant $\lambda =2\sum_{q}\alpha_{q}^{2}\omega_{q}^{-1}$ (*23*), giving

$$\lambda =\frac{\epsilon_{0}\hbar \Omega_{p}^{2}}{2\pi k_{B}}\frac{d\rho }{dT}+\Delta \lambda$$ (2)

where additional contributions to λ from phonon modes with $\hbar \omega_{q}>>k_{B}T$ are denoted by Δλ. Hence, the steep gradient of the resistivity at low temperatures is indicative of strong electron-phonon coupling.

This procedure for estimating λ has been demonstrated to work well for the elements, giving values of λ within 10% of the experimental values obtained from tunneling experiments (*24*). Using the low-temperature resistivity gradient *d*ρ/*dT* = 0.9 μΩcm K^−1^ and $\hbar \Omega_{p}≃3.5\;\text{eV}$ from our ab initio calculations (Supplementary Materials), we obtain λ ≃ 2.75 (Table 1). This represents one of the highest values of λ ever reported in an element, which, together with the range of the *T*-linear resistivity to low temperatures, suggests significant phonon spectral weight at very low energies. This rather unusual phonon spectrum may also cause the anomalous *T* dependence of the upper critical field discussed above.

**Table 1:** Experimental and calculated material parameters in Bi-III and in the reference materials In_5_Bi_3_ and Ca_3_Rh_4_Sn_13_ (see text). The coherence length ξ_exp_ is obtained from the upper critical field and can be compared to ξ_calc_ = *ℏv*_F_/(πΔ). The Fermi velocity *v*_F_ is estimated by scaling the density functional theory (DFT) estimate $v_{F}^{0}$ by (1 + λ_ρ_)^−1^, where λ_ρ_, the electron-phonon coupling constant obtained from the slope of ρ(*T*) with the help of a DFT estimate of the plasma frequency Ω_p_ (see text), is listed separately. In In_5_Bi_3_ and Ca_3_Rh_4_Sn_13_, it can be compared to the ratio of measured and calculated Sommerfeld coefficients λ_C_ = γ_exp_/γ_DFT_−1. In Bi-III, no heat capacity data are as yet available. The gap size 2Δ = η*k*_B_*T*_c_ with η = 5.7 for Ca_3_Rh_4_Sn_13_ (*27*) and values ≃ 5 and 4 assumed for Bi-III and In_5_Bi_3_ (Supplementary Materials). n/a, not applicable.

	***T*_c_** **(K)**	***B*_c2_** **(T)**	**ξ_exp_** **(Å)**	**ξ_calc_** **(Å)**	**Ω_p_** **(eV)**	**λ_ρ_**	**λ_C_**
Bi-III	7.05	2.45	116	172	3.5	2.75	n/a
In_5_Bi_3_	4.14	0.3	331	500	2.5	1.25	0.90
Ca_3_Rh_4_Sn_13_	8.0	4.0	91	126	2.2	1.1	0.80

## DISCUSSION

The origin of additional phonon spectral weight at very low energy may be sought in the abovementioned phason or sliding mode, which results naturally from the incommensurate host-guest structure of Bi-III. Our ab initio phonon calculations (Fig. 4B) indeed show that two such low-lying modes are to be expected in Bi-III, because there are two chains per primitive unit cell in the 42-atom approximant, and that they would make a major contribution to λ. The dispersion is strong along the chain direction (*c*), but because the chains are only weakly coupled to each other, the sliding modes have an almost flat dispersion perpendicular to *c*. There is therefore an extended region of *q* space where ω_*q*_ is strongly reduced, producing effectively a one-dimensional phonon dispersion. Such a nearly flat dispersion in directions perpendicular to the incommensurate axis has recently been observed with neutron scattering in the incommensurate spin ladder compound Sr_14_Cu_24_O_41_ (*25*). Because the approximant structures used in numerical calculations are necessarily commensurate, the phason modes in Fig. 4B are shifted to finite frequency at *q* = 0 and hybridize with the conventional acoustic modes at low *q*, but this does not change the central conclusion, namely, that the sliding modes contribute significant spectral weight at low frequency to the phonon spectrum. This results in an enhanced value of the electron-phonon coupling strength, $\lambda \sim \sum_{q}\omega_{q}^{-1}$, if we ignore the effects of anharmonicity, manifested in the rapidly saturating ρ(*T*), disorder, which could pin the phason modes, and damping, which could become relevant for low phonon frequencies.

Table 1 lists key parameters for Bi-III and compares them to those of the structurally similar but commensurate In_5_Bi_3_ and the quasi-skutterudite system (Sr/Ca)_3_Rh_4_Sn_13_. In_5_Bi_3_ is also a type II superconductor, with *T*_c_ = 4.14 K, showing similar resistivity saturation (*26*). In (Sr/Ca)_3_Rh_4_Sn_13_, a second-order structural transition associated with a soft phonon branch can be continuously suppressed to zero temperature by varying composition or pressure, resulting in a *T*-linear resistivity at low temperatures, pronounced negative curvature of ρ(*T*), and superconductivity with *T*_c_ ≈ 8 K (*27*). A similar argument applies for (Sr/Ca)_3_Ir_4_Sn_13_, which is also a strong coupling superconductor (*28*, *29*). Moreover, a comparison can be made with amorphous bismuth (Bi-a) (*30*, *31*), which superconducts below 6.15 K and has *B*_c2_ ≃ 2.6 T (*32*). These similarities to Bi-III suggest that peculiarities of the phonon spectrum, including prominent low-energy modes, are driving strong coupling superconductivity in both cases. Superconductivity with *T*_c_ ~ 4 K is also found in other elemental host-guest structures such as Sb-II and Ba-IV (*33*–*35*), and, like Bi-III, they may deserve closer examination.

Enhanced critical fields, a steep increase in the electrical resistivity with *T*, and resistivity saturation occur more widely in materials with strong electron-phonon coupling and low-energy phonon modes, such as the A15 superconductors (*36*). Our findings suggest that incommensurate structures offer a new approach for generating such low-lying spectral weight. Bi-III can also be considered in the context of other and arguably more complex materials that share a linear *T* dependence of the resistivity (*37*) with a similar slope. At much higher pressures than that required to reach the Bi-III phase, As, Sb, Ba, Sr, Sc, K, and Rb also assume incommensurate host-guest structures [(*38*, *39*) and references therein]. Moreover, recent findings in the spin ladder compound Sr_14_Cu_24_O_41_ (*25*) demonstrate the existence of quasiperiodic structures in compounds at ambient pressure and their potential for new forms of magnetic as well as structural frustration. Very little is so far known about the electronic and vibrational excitations of quasiperiodic systems. Bi-III and other incommensurate host-guest structures open up a new field of research on the boundary between conventional crystallinity and disorder.

## MATERIALS AND METHODS

### High-pressure measurements

Bismuth samples were extracted from a large single crystal obtained commercially (5N Bi, residual resistance ratio ≃ 100; MaTecK). Samples were mounted in a piston-cylinder pressure cell (*40*) for four-point ac electrical resistivity measurements to pressures exceeding 31 kbar, using Daphne oil 7373 as a pressure medium and the *T*_c_ of Sn as a manometer (*41*). Low-temperature measurements were performed in a Quantum Design Physical Property Measurement System and in an adiabatic demagnetization refrigerator, and resistivity data were scaled at 300 K to published values (*42*). The magnetization was measured in a Cryogenic SQUID magnetometer up to 96 kbar using an ultralow background CuTi moissanite anvil cell, with glycerol as a pressure medium and ruby fluorescence at room temperature for pressure determination (*43*).

### DFT calculations

The electronic structure was calculated using the generalized gradient approximation (*44*) with WIEN2k (*45*), in the 32-atom approximant structure that is most closely related to the Bi-III host-guest lattice (*15*), and checked for consistency with other approximants (Supplementary Materials). Using the experimentally determined pressure dependence of the host lattice unit cell volume, the measured lattice parameters for Bi-III at 6.8 GPa (*15*) were converted into the expected lattice parameters at 3 GPa, which is the approximate pressure of our measurements (*a* = 8.671 Å and *c* = 12.717 Å). The product of the smallest atomic sphere radius and the largest *k* vector of the plane wave expansion of the wave function, *Rk*_max_, was set to 8, and 10,000 *k* points were used. Spin orbit coupling was included without relativistic local orbitals, and the plasma frequency was calculated with the WIEN2k package Optic (*46*), which averages the computed squared momenta for all the bands over the Fermi surface

$$\Omega_{x}^{2}=\frac{e^{2}}{4\pi \epsilon_{0}}\frac{1}{\pi^{2}m^{2}}\sum_{n}\int d^{3}kp_{x;n,k}^{2}\delta (\epsilon_{n,k}-\epsilon_{F})$$ (3)

and, likewise, for the other principal axes *y* and *z*. Here, **p**_*n*,*k*_ is the momentum expectation value for states in band *n* with crystal momentum **k**. The overall plasma frequency is estimated by averaging the squared frequencies, $\Omega_{p}^{2}=\frac{1}{3}(\Omega_{x}^{2}+\Omega_{y}^{2}+\Omega_{z}^{2})$. The Bi-III phonon spectrum was obtained with the CASTEP package (*47*), using an optimized 42-atom approximant structure (*48*) and finite differences in conjunction with nondiagonal supercells (*49*), with a coarse *q* point grid of up to 8 × 8 × 8.

## Supplementary Material

http://advances.sciencemag.org/cgi/content/full/4/4/eaao4793/DC1

## SUPPLEMENTARY MATERIALS

Supplementary material for this article is available at http://advances.sciencemag.org/cgi/content/full/4/4/eaao4793/DC1 section S1. Material parameters from DFT section S2. Characterization of bismuth crystal section S3. *B*_c1_ from high-pressure magnetization data fig. S1. Comparison between Bi-III approximants. fig. S2. Results of DFT calculations in Bi-III, including electronic density of states, plasma frequencies, and phonon density of states. fig. S3. Experimental observations in the reference material In_5_Bi_3_. fig. S4. X-ray characterization of bismuth sample. fig. S5. Extracting estimates of the lower critical field from high-pressure zero field–cooled magnetization measurements in Bi-III. References (*50*–*54*)
