# Supplementary material for: Strong coupling superconductivity in a quasiperiodic host-guest structure
Source: Sci Adv. 2018 Apr 13;4(4):eaao4793. doi: 10.1126/sciadv.aao4793 (PMC5898833; doi:10.1126/sciadv.aao4793)
Supplement: http://advances.sciencemag.org/cgi/content/full/4/4/eaao4793/DC1 [file supp_4_4_eaao4793__index.html]

Science Advances | Science Advances

## Supplementary Materials

**This PDF file includes:**

- section S1. Material parameters from DFT
- section S2. Characterization of bismuth crystal
- section S3. *B*c1 from high-pressure magnetization data
- fig. S1. Comparison between Bi-III approximants.
- fig. S2. Results of DFT calculations in Bi-III, including electronic density of states, plasma frequencies, and phonon density of states.
- fig. S3. Experimental observations in the reference material In5Bi3.
- fig. S4. X-ray characterization of bismuth sample.
- fig. S5. Extracting estimates of the lower critical field from high-pressure zero field–cooled magnetization measurements in Bi-III.
- References (*50–54*)

Download PDF

**Files in this Data Supplement:**

- Adobe PDF - aao4793\_SM.pdf
