# Supplementary material for: Strong coupling superconductivity in a quasiperiodic host-guest structure
Source: Sci Adv. 2018 Apr 13;4(4):eaao4793. doi: 10.1126/sciadv.aao4793 (PMC5898833; doi:10.1126/sciadv.aao4793)
Supplement: http://advances.sciencemag.org/cgi/content/full/4/4/eaao4793/DC1 [file aao4793_SM.pdf]

Science Advances

AAAS

advances.sciencemag.org/cgi/content/full/4/4/eaao4793/DC1

Supplementary Materials for

Strong coupling superconductivity in a quasiperiodic host-guest structure

Philip Brown, Konstantin Semeniuk, Diandian Wang, Bartomeu Monserrat, Chris J. Pickard, F. Malte Grosche

Published 13 April 2018, *Sci. Adv.* **4**, eaao4793 (2018)
DOI: 10.1126/sciadv.aao4793

This PDF file includes:

- section S1. Material parameters from DFT
- section S2. Characterization of bismuth crystal
- section S3. B_{c1} from high-pressure magnetization data
- fig. S1. Comparison between Bi-III approximants.
- fig. S2. Results of DFT calculations in Bi-III, including electronic density of states, plasma frequencies, and phonon density of states.
- fig. S3. Experimental observations in the reference material In_5Bi_3.
- fig. S4. X-ray characterization of bismuth sample.
- fig. S5. Extracting estimates of the lower critical field from high-pressure zero field–cooled magnetization measurements in Bi-III.
- References (*50*–*54*)

Supplementary Material for
"Strong coupling superconductivity in a quasiperiodic host-guest structure"

This material elaborates on the extraction of parameters displayed in Table 1 in the main manuscript, from numerical calculations within density functional theory (DFT) and from electronic transport measurements. It gives further information about the bismuth sample and its characterization and it provides technical details on the interpretation of high pressure magnetization measurements in Bi-III and how they were used to extract estimates of the lower critical field B_{c1}.

section S1. Material parameters from DFT

Electronic structure calculations were carried out for a variety of commensurate lattice structures approximating the actual incommensurate Bi-III structure. The approximants all feature a bct guest unit cell, but the total number of atoms per unit cell varies between 22 and 54. The lattice constant ratio $c_{\mathrm{H}}/c_{\mathrm{G}}$ is 4/3 for the 32-atom approximant Bi-IIIa, differing by less than 2% from the incommensurate ratio, which is $\simeq 1.309$ (fig. S1). We concentrated on the 32-atom approximant in calculations of the electronic density of states and plasma frequency, but checked for robustness against choice of approximant. With $Rk_{\mathrm{max}} = 8$ (product of the smallest atomic sphere radius and the largest k-vector of the plane wave expansion of the wavefunction), $10,000$ k-points and including spin-orbit coupling without relativistic local orbitals, Wien2k calculations using PBE-GGA gave a density of states at the Fermi energy of 284.30 Ryd^{-1} per 32-atom unit cell or 0.653/eV per Bi atom, consistent with results reported in [13] (fig. S2). Using a unit cell volume of 955.2 $\mathrm{\mathring{A}}^3$ at 30 kbar, which is extracted from experimental data reported in [15], the density of states per unit volume is $0.022/(\mathrm{eV}\ \mathrm{\mathring{A}}^3)$.

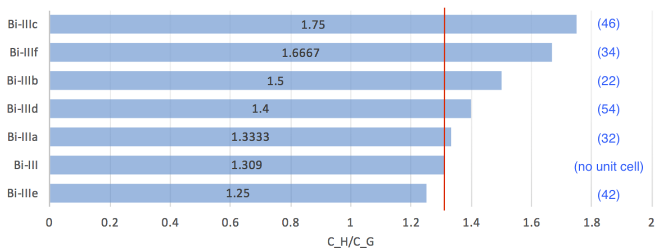

fig. S1. **Comparison between Bi-III approximants.** Bar graph comparing the host/guest lattice constant ratio $c_{\mathrm{H}}/c_{\mathrm{G}}$ for various approximants to that of the Bi-III structure (red vertical line). Numbers in parentheses denote the number of atoms in the unit cell.

Estimates of the plasma frequency in Bi-III

Using the OPTIC package [46], it is furthermore possible to extract estimates of the plasma frequency Ω_p for different crystallographic directions. Because this calculation involves integrals over $k-$states which are dominated by contributions close to the Fermi surface, high resolution in $k-$space is important. Varying the number of $k-$points showed that the averaged plasma frequency is robust at $\Omega_p = \sqrt{(2\Omega_x^2 + \Omega_z^2)/3} \simeq 3.5$ eV/$\hbar$ (fig. S2). Using Ω_p and the measured slope of the electrical resistivity at low T, $d\rho/dT \simeq 0.9\mu\Omega$cm, we arrive at the estimate $\lambda \simeq \lambda_\rho = \frac{\epsilon_0 \hbar \Omega_p^2}{2\pi k_B}\frac{d\rho}{dT} \simeq 2.75$. Phonon dispersion calculations described in the main text (Fig. 4) suggest that this unusually large electron-phonon coupling can be attributed to two low-lying phonon modes in the 42-atom approximant used for that calculation. Numerical estimates of their contribution to λ are problematic, because in the ideal incommensurate structure, these modes should have zero frequency at $q = 0$, whereas they are pulled to about 0.8 meV in the approximant structure, and the contribution to λ is scaled by $1/\omega$. Our calculations (see also fig. S2) show a combined contribution of the two phason modes at the zone centre and at $(0, 0.5, 0)$ of 0.23 and 0.21, respectively, whereas the largest contribution from any of the remaining phonon branches is 0.013 and 0.012 at either reciprocal space position, illustrating the enhanced electron-phonon coupling from these two modes, which would be further boosted as the mode frequency is lowered in the actual, incommensurate structure.

Estimates of superconducting parameters in Bi-III

Combining the density of states per unit volume and the plasma frequency, we can obtain a DFT estimate for the Fermi velocity v_F^0 via $\langle (v_F^0)^2 \rangle = \frac{3\epsilon_0}{e^2}\frac{\Omega_p^2}{g(E_F)}$ (e.g. [50]) as $v_F^0 \simeq 4.65 \times 10^5$ m/s. Using this and the estimated λ, we can find the renormalised Fermi velocity $v_F = v_F^0/(1+\lambda)$, which in turn enters the estimate of the coherence length $\xi = \frac{\hbar v_F}{\pi \Delta}$. To estimate the gap Δ in a strong-coupling superconductor, we can first of all use the modified McMillan formula as proposed by Allen and Dynes [51]

$$k_B T_c = \frac{\hbar \omega_{ln}}{1.2} \exp^{-\frac{1.04(1+\lambda)}{\lambda - \mu^*(1+0.62\lambda)}} \qquad (4)$$

to extract the logarithmically averaged phonon frequency ω_{ln} from T_c and λ, approximating $\mu^* \simeq 0.1$, which is relatively unimportant as $\lambda >> \mu^*$ in Bi-III. We find

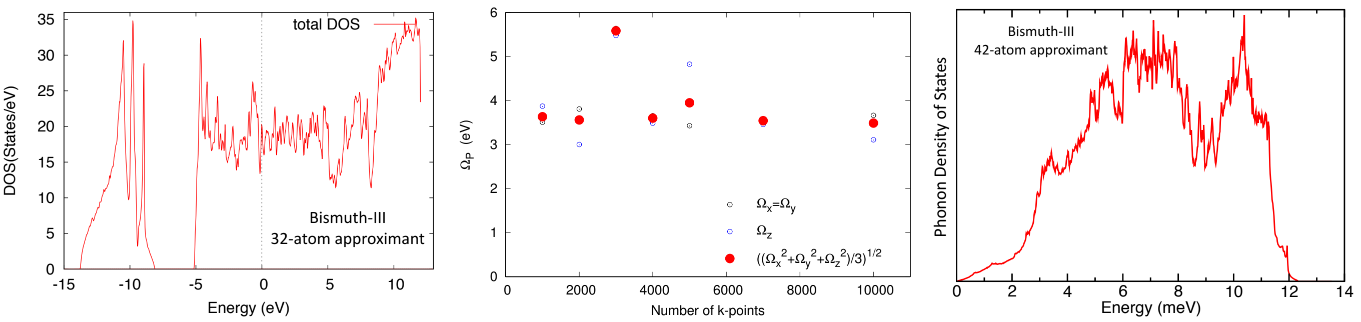

fig. S2. **Results of DFT calculations in Bi-III.** Left: Energy dependence of the density of states per conventional unit cell for the 32-atom approximant computed with Wien2k. Middle: Plasma frequencies of Bi-III 32-atom approximant for increasing resolution in reciprocal space. Although the extracted frequencies vary significantly at low numbers of $k-$points, the averaged plasma frequency $\Omega_p = \sqrt{(2\Omega_x^2 + \Omega_z^2)/3}$ is steady at about 3.5 eV for more than 5000 $k-$points. Right: Phonon density of states calculated in a coarse $4 \times 4 \times 4$ grid for the 42-atom approximant. The high frequency optical modes dominate the figure, but the tail below 2 meV – which does not follow the ω^2 form that would be expected for acoustic phonons – indicates the contribution from the two low-lying modes, which in this approximant start at about 0.8 meV. In the actual, incommensurate structure of Bi-III, this tail would be expected to reach down to zero frequency. The contribution to λ is weighted by $1/\omega$, giving enhanced importance to the low energy end of the phonon spectrum.

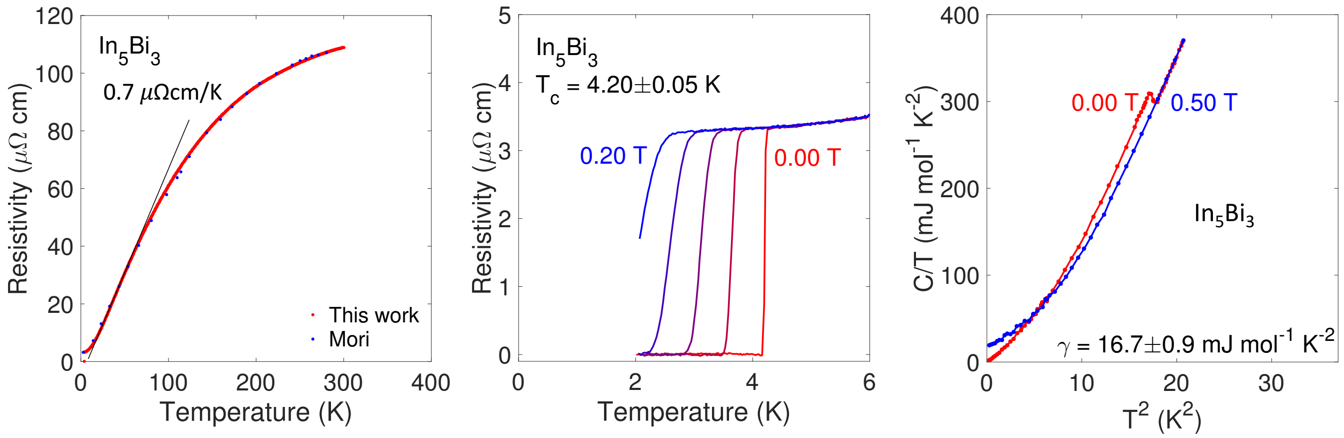

fig. S3. **Experimental observations in the reference material In_5Bi_3.** (Left) Electrical resistivity vs. T in In_5Bi_3, including previous data from [26] for comparison. The maximum slope $d\rho/dT \simeq 0.7\mu\Omega$cm. (Middle) Resistive superconducting transition, showing $T_c \simeq 4.2$ K, with an upper critical field $B_{c2} \simeq 0.3$ T. (Right) Low temperature heat capacity, showing the superconducting anomaly and the normal state Sommerfeld coefficient $\simeq 17$ mJ/(mol K^2).

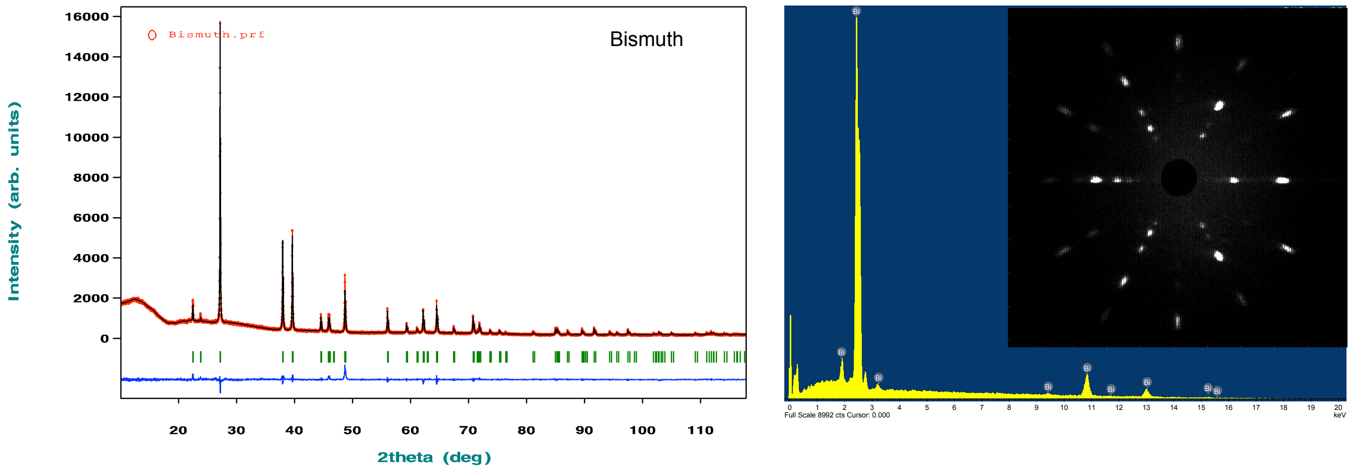

fig. S4. **X-ray characterization of bismuth sample.** Powder x-ray pattern (left), EDX spectrum (right) and Laue pattern (right inset) taken on the bismuth crystal from which the sample for this study was extracted.

$\hbar\omega_{ln}/k_B \simeq 41$ K. This is much larger than the expected lowest energies of the sliding mode, because it represents an average over the entire phonon spectrum, which includes substantial spectral weight at energies of up to 12 meV. Using this value of ω_{ln} in Carbotte's empirical relation $2\Delta/(k_BT_c) = 3.53\left(1 + 12.5\left(\frac{k_BT_c}{\hbar\omega_{ln}}\right)^2 \ln\frac{\hbar\omega_{ln}}{2k_BT_c}\right)$ [52] we obtain $2\Delta/(k_BT_c) \simeq 4.93$, consistent with the experimental values in Fig. 31 of [52]. Combining the results for$\Delta$and v_F finally produces the estimate of the superconducting coherence length $\xi \simeq 172$ Å given in the main article.

Estimates relevant to In_5Bi_3

Applying the same methodology to the related material In_5Bi_3 gives $g(E_F) = 0.47$/eV per atom or $0.017/(\text{eV Å}^3)$ per unit volume, $\hbar\Omega_p \simeq 2.68$ eV, $v_F^0 \simeq 4.1 \times 10^5$ m/s, and a DFT value for the Sommerfeld coefficient $\gamma_0 = 9$ mJ/(mol K^2). We have experimentally determined the Sommerfeld coefficient to be $\gamma_{exp} = 17$ mJ/mol K^2 (fig. S3), suggesting that the electron-phonon coupling constant in In_5Bi_3 is $\lambda_C = \gamma_{exp}/\gamma_0 - 1 \simeq 0.9$. We have also measured $\rho(T)$ of In_5Bi_3 and found that it agrees with earlier reports [26] and has a maximum slope near 30 K of $d\rho/dT \simeq 0.7\mu\Omega$cm, which together with the calculated Ω_p gives $\lambda \simeq 1.25$, somewhat higher than the estimate based on the heat capacity. As in Bi-III, we can use this to extract $\omega_{ln} \simeq 44$ K and $2\Delta/(k_BT_c) \simeq 4.2$, and finally $\xi \simeq 500$ Å.

Estimates relevant to $Ca_3Rh_4Sn_{13}$

The same procedure was carried out in the case of $Ca_3Rh_4Sn_{13}$. We find $g(E_F) = 0.031/(\text{eV Å}^3)$ and $\hbar\Omega_p \simeq 2.2$ eV, giving $v_F^0 \simeq 2.45 \times 10^5$ m/s and $\gamma_0 = 33.4$ mJ/(mol K^2). Comparing this to the experimental value [53] of 60 mJ/(mol K^2) gives an estimate for λ as $\lambda_C = 0.8$, whereas the Sommerfeld coefficient 75 mJ/(mol K^2) reported in [54] would produce $\lambda_C = 1.24$. The electrical resistivity reported in the supplemental material of [27] suggests $d\rho/dT \simeq 0.88$ $\mu\Omega$cm/K, giving $\lambda_\rho = 1.05$. We use this value to extract $\omega_{ln} \simeq 104$ K and $2\Delta/(k_BT_c) \simeq 4.0$. This contrasts with the value $2\Delta/(k_BT_c) \simeq 5.5$ determined in [27] by analysing heat capacity data. The latter value together with the renormalised v_F gives $\xi \simeq 130$ Å, whereas the former gives $\xi \simeq 180$ Å.

Finally, we can estimate the superconducting penetration depth as $\ell_\lambda = \frac{(1+\lambda)^{1/2}c}{\Omega_p}$, where c is the speed of light. This gives a penetration depth of about 109 nm for Bi-III and similar values for the other two materials.

section S2. Characterization of bismuth crystal

The crystal used in this study was produced by Bridgman growth from 5N bismuth by the crystal growth company Mateck (www.mateck.com). It was characterised by Laue and powder diffraction as well as EDX analysis (fig. S4).

section S3. B_{c1} from high-pressure magnetization data

We can extract estimates for the lower critical field B_{c1} from measurements of the T-dependence of the magnetization M under high pressure (fig. S5). These estimates should be seen as upper limits on the intrinsic $B_{c1}(0)$, because considerable corrections may arise from pinning effects and from the demagnetizing fields caused by the platelet-shaped sample, which is oriented perpendicular to the applied field. The foot of the magnetization step associated with the superconducting transition in zero-field-cooled data, T_{c1}, indicates the temperature at which full flux expulsion makes way to the mixed state. In moderate applied fields, this shifts towards lower T rapidly, making it possible to construct a plot of $B_{c1}(T)$. The high T end of the transition, labelled T_c in the figure, remains almost unchanged, as expected in a type II superconductor (fig. S5). A fit to the empirical form $B_{c1}(T) = B_{c1}(0)(1-(T/T_c)^2)$ yields an estimated lower critical field $B_{c1}(0) \approx 12$ mT at pressures between 30 and 40 kbar. With increasing pressure, estimates for $B_{c1}(0)$ change, ranging between 10 mT and 20 mT.

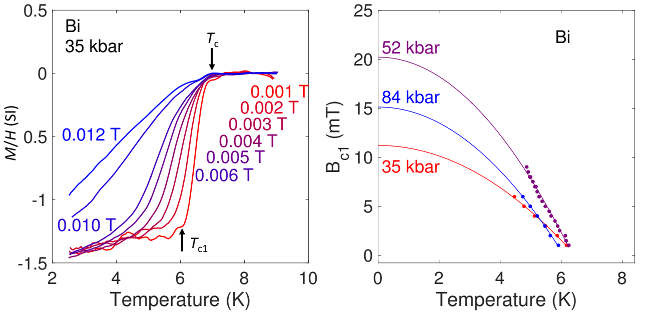

fig. S5. **Extracting estimates of the lower critical field from high pressure magnetization measurements in Bi-III.** (Left:) zero-field-cooled magnetization traces at different applied fields, showing the shift of the foot of the transition at T_{c1}. (Right:) tracking this shift, we can construct the T-dependence of B_{c1}. Extrapolating this to $T = 0$ gives estimates of the low temperature B_{c1} which range between 10 mT and 20 mT, depending on the pressure.
